# LncRNA WT1-AS downregulates lncRNA UCA1 to suppress non-small cell lung cancer and predicts poor survival

**DOI:** 10.1186/s12885-020-07767-4

**Published:** 2021-01-29

**Authors:** Yanhui Wan, Da Yao, Fuyuan Fang, Youyu Wang, Guodong Wu, Youhui Qian

**Affiliations:** Department of Thoracic Surgery, Shenzhen Second People’s Hospital, The First Affiliated Hospital of Shenzhen University, No.3002, Sungang west road, Futian district, Shenzhen City, 518000 People’s Republic of China

**Keywords:** Non-small cell lung cancer, WT1-AS, UCA1, Prognosis

## Abstract

**Background:**

LncRNA WT1-AS inhibits gastric cancer, while its role in other cancers is unknown. We investigated the role of WT1-AS in non-small cell lung cancer (NSCLC).

**Methods:**

Sixty-six NSCLC patients (40 males and 26 females; 36 to 68 years old; mean age 52.7 ± 6.4 years old) were selected from the 178 NSCLC patients operated on for lung cancer between 2010 and 2013. RT-qPCR was used to analyze the expression of lncRNA. Overexpression experiments were performed to assess interactions between lncRNAs. CCK-8 assay was carried to evaluate the roles of WT1-AS and UCA1 in regulating cell proliferation. Cell invasion and migration assays were performed to assess the roles of WT1-AS and UCA1 in regulating cell invasion and migration. Western-blot was performed to illustrate the effect of WT1-AS and UCA1 in EMT.

**Results:**

WT1-AS was downregulated in NSCLC and was correlated with poor survival. The expression of WT1-AS in NSCLC was not correlated with clinical stages. LncRNA UCA1 was upregulated in cancer tissues and inversely correlated with WT1-AS. Overexpression of UCA1 did not affect WT1-AS, while overexpression of WT1-AS led to inhibited expression of UCA1. Overexpression of UCA1 resulted in increased proliferation, EMT, migration and invasion of NSCLC cells, while overexpression of WT1-AS showed opposite effects. In addition, overexpression of UCA1 inhibited the role of overexpression of WT1-AS.

**Conclusions:**

Therefore, overexpression of WT1-AS may inhibit the cell proliferation and EMT to decrease cell migration and invasion of NSCLC cells by downregulating UCA1.

**Supplementary Information:**

The online version contains supplementary material available at 10.1186/s12885-020-07767-4.

## Background

Lung cancer has the highest incidence of all cancers worldwide [1]. Due to its aggressive nature, lung cancer is also responsible for a large number of cancer-related mortalities [2]. Non-small cell lung cancer (NSCLC) accounts for 85% of all lung cancer cases [3]. Clinical treatment of NSCLC is challenged by the fact that more than 70% of NSCLC patients are diagnosed at advanced or middle stages, with a low 5-year overall survival rate of less than 15% [4]. Smoking is closely correlated with NSCLC, while some never-smokers can also develop NSCLC, indicating the important roles of genetic alterations in NSCLC [5].

Accumulative studies have revealed lncRNAs (> 200 nt) are not “noise” or “background” of the transcriptome [6]. Instead, lncRNAs have critical functions in diverse cellular processes, including the development of different types of human cancer [7, 8]. Although lncRNAs do not encode proteins, they regulate the expression of genes at multiple levels, thereby participating in cancer biology [9]. Regulating the expression of cancer-related lncRNAs provides new insights into the development of cancer therapies [10]. Decreased expression levels of WT1-AS are closely correlated with the progression of gastric cancer [11], indicating its tumor-suppressive role in this disease. However, the function of WT1-AS in other types of cancer remains unknown. UCA1 is a well-studied oncogenic lncRNA in many types of cancer including NSCLC [12]. We performed a whole-genome lncRNAs analysis and found an inverse correlation between WT1-AS and UCA1 in NSCLC (data not shown). Therefore, this study was carried out to investigate the interaction between WT1-AS and UCA1 in NSCLC.

## Methods

### Patients and specimens

We selected 66 NSCLC patients (40 males and 26 females; 36 to 68 years old; mean age 52.7 ± 6.4 years old) selected from the 178 NSCLC patients admitted by Shenzhen Second People’s Hospital from June 2010 to June 2013. The 66 NSCLC patients included 30 cases of adenocarcinoma, 25 cases of squamous cell carcinoma and 11 cases of large cell carcinoma. All patients were smokers or had a previous history of smoking. No exposure to asbestos and other carcinogens was observed. A family history of lung cancer was not observed. Inclusion criteria: 1) patients received histopathological diagnosis; 2) all patients were newly diagnosed cases; 3) no initiated therapy. Exclusion criteria: 1) patients transferred from another hospital (45 cases excluded); 2) other clinical disorders were diagnosed (64 cases excluded, including 12 cases of heart disease, 26 cases of diabetes, 1 case of skin cancer, 17 cases of severe infection, 2 cases of bone fracture, 1 case of metal disorder, 2 cases of renal injury, 1 case of cystitis, and 2 cases of glaucoma); 3) patients with a history of malignancies (3 cases excluded). Based on clinical findings, the 66 patients included 12, 10, 22 and 22 cases at stage I-IV (AJCC), respectively. Written informed consent was signed by all patients. This study was approved by the Ethics Committee of the aforementioned hospital. Lung biopsy was performed to diagnose NSCLC. During lung biopsy, NSCLC and adjacent normal (non-cancer) tissues were obtained and stored in − 80 °C. All biopsies were completed under the guidance of MRI before the initiation of therapies. All tissue samples were subjected to histopathological exams and all NSCLC tissues contained more than 90% cancer cells and all non-tumor tissues contained less than 2% cancer cells.

### A 5-year follow-up

After admission, the 66 NSCLC patients were monitored for 5 years through phone calls and/or outpatient visits every 1–2 months. Patients died of causes unrelated to NSCLC were excluded.

### NSCLC cells and transient transfection

Two human NSCLC cell lines H1993 and H1581 (ATCC, USA) were used. RPMI-1640 medium (90%) was mixed with FBS (10%) to grow cells. Cells were grown at 37 °C in an incubator with 95% humidity and 5% CO2. WT1-AS or UCA1 overexpression vector was constructed with pcDNA3 vector (GenePharma, Shanghai, China). At the confluence of 70–90%, H1993, and H1581 cells were harvested and transfected with either 10 nM WT1-AS or UCA1 overexpression vector. Negative control (NC) experiment was performed by transfecting empty vector into the same number of cells. To perform Control (C) cells, cells without transfections were grown until the end of experiments. After cell transfections, cells were cultivated under normal conditions for another 24 h.

### RT-qPCR

RNAzol (Sigma-Aldrich, USA) was used for RNA isolation from H1993 and H1581 cells (10^6^ cells) or tissues (about 0.025 g tissues in 0.5 ml RNAzol reagent). DNase I digestion was performed on all RNA samples. Following that, reverse transcriptions were performed to synthesize cDNAs. With cDNAs as a template, qPCRs were performed to detect the expression of WT1-AS and UCA with 18S rRNA as the endogenous control. Three technical replicates were included in each experiment and Ct values of targeted genes were normalized to 18S rRNA using the 2^-ΔΔCT^ method. The primer sequences were: WT1-AS Forward: 5′-GAGGACAGA GAGGCATGGAG-3′; Reverse: 5′-ACCCCTAGGCAAGGAGAAGA-3′; UCA1 Forward: 5′-ACGCTAACTGGCACCTTGTT-3′; Reverse: 5′-TGGGGATTACTGGGGTAGGG-3′; 18S rRNA Forward: 5′-CGGCTACCACATCCAAGGAA-3′; Reverse: 5′-TGTCACTACCTCCCCGTGTCA-3′.

### Cell invasion and migration assays

H1993 and H1581 cells were transferred to the upper Transwell chamber (8 μm pore, Corning, Corning, NY) with 3000 cells in 0.1 ml non-serum medium per well. Each cell transfection group included three replicates. The lower Transwell chamber was filled with medium supplemented with 20% FBS. Matrigel (356,234, Millipore, USA)–coated membrane was used in invasion assay, while uncoated membranes were used in migration assay. Cells were cultured under the condition of 37 °C and 5% CO_2_ for 12 h. After that, upper surface was cleaned, and 0.5% crystal violet (Sigma-Aldrich, USA) was used to stain lower surface at 25 °C for 30 min. An optical microscope was used to observe the stained cells. All groups were normalized to the C group, which was set to “100”.

### Cell proliferation assay

Cell count Kit-8 (CCK-8, Sigma-Aldrich) was used to evaluate the effect of overexpression of WT1-AS and UCA1 on proliferation of H1993 and H1581 cells. Briefly, 3× 10^4^ cells were mixed with 1 mL medium. Under the above conditions, cells were grown with a cell culture plate (96 Wells, 0.1 mL per well) at 4 h before the end of cell culture, cck-8 solution (10 μL) was added to each well. Then 10 μL DMSO was added, and OD values at 450 nm were measured.

### Western-blot

H1993 and H1581 cells were mixed with RIPA solution (Beyotime) to extract total proteins. The protein concentration was detected by the BCA assay kit (Beyotime). All protein samples were denatured by incubating the samples in boiling water for 15 min. After that, electrophoresis was performed using 10% SDS-PAGE gel to separate protein molecules. After that, proteins were transferred to PVDF membranes, followed by blocking in PBS containing 5% non-fat milk at room temperature for 2 h. After that, membranes were incubated with rabbit primary antibodies of E-cadherin (ab40772, Abcam), vimentin (ab92547, Abcam), p53 (ab131442, Abcam) and β-actin (ab8226, Abcam) at 4 °C for 18 h. After that, membranes were further incubated with IgG-HRP goat anti rabbit (MyBioSource) at 24 °C for 2 h. Signals were developed using ECL (Sigma-Aldrich) and quantity one was used to normalize the signals.

### Statistical analysis

Data from at least 3 biological replicates of each experiment were used to calculate mean values. One-sided t test and Chi square test were performed to explore differences between two types of (NSCLC and non-cancer) tissues. ANOVA Tukey’s test was used to compare multiple groups. Correlations were explored using linear regression. Based on WT1-AS expression in NSCLC tissues, 66 NSCLC patients were grouped into low and high WT1-AS level groups, 34 and 32 cases in each group (Youden’s index at the cutoff value), respectively. Survival data of both groups were used to plot survival curves, followed by comparison by log-rank test. *p* < 0.05 were considered as statistically significant.

## Results

### WT1-AS was downregulated in NSCLC but not affected by clinical stages

**Fig. 1:**
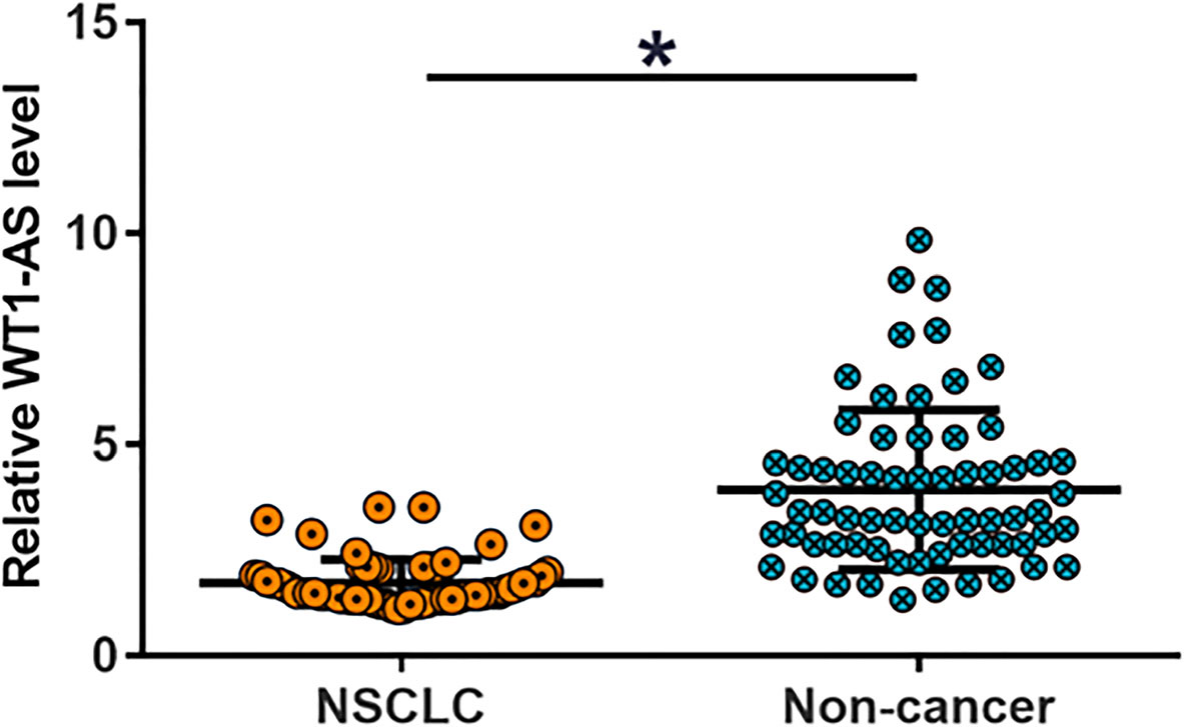
WT1-AS was downregulated in NSCLC WT1-AS expression levels measured by RT-qPCR and compared by paired t-test, *, *p* < 0.05

The expression of WT1-AS in two types of tissues (NSCLC and non-cancer) from NSCLC patients (*n* = 66) was detected by RT-qPCR. It was observed that the expression levels of WT1-AS were significantly lower in NSCLC tissues compared to that in non-cancer tissues (Fig. 1, *p*< 0.05). Based on clinical findings, the 66 patients included 12, 10, 22 and 22 cases at stage I-IV (AJCC), respectively. The chi-squared test showed that the expression levels of WT1-AS and UCA1 in were not significantly correlated with the clinical stage (Tables 1 and 2, *p*< 0.05). Moreover, no significant differences in the expression levels of WT1-AS and UCA1in either non-tumor tissues or NSCLC tissues were observed among different subtypes of NSCLC (data shown in Table 1 and Table 2).

**Table 1:** Association with WT1-AS and the clinical pathological characteristics of NSCLC patients

	Group	Cases	High	Low	χ^2^	*P* value
**histological classification**	squamous	25	10	11	0.54	*0.76*
	adenocarcinoma	30	14	15		
	large cell carcinoma	11	4	7		
**Stage**					2.46	*0.45*
	I	12	4	8		
	II	10	6	4		
	III	22	10	12		
	IV	22	13	9		

For analysis of the association between WT1-AS levels and clinical features, Pearson’s χ2 tests were used *P< 0.05

**Table 2:** Association with UCA1 and the clinical pathological characteristics of NSCLC patients

	Group	Cases	High	Low	χ^2^	*P* value
**histological classification**	squamous	25	11	14	1.33	*0.51*
	adenocarcinoma	30	17	13		
	large cell carcinoma	11	6	5		
**Stage**					0.86	*0.84*
	I	12	5	7		
	II	10	4	6		
	III	22	12	10		
	IV	22	11	11		

For analysis of the association between UCA1 levels and clinical features, Pearson’s χ2 tests were used *P< 0.05

### The expression of WT1-AS predicted NSCLC survival

**Fig. 2:**
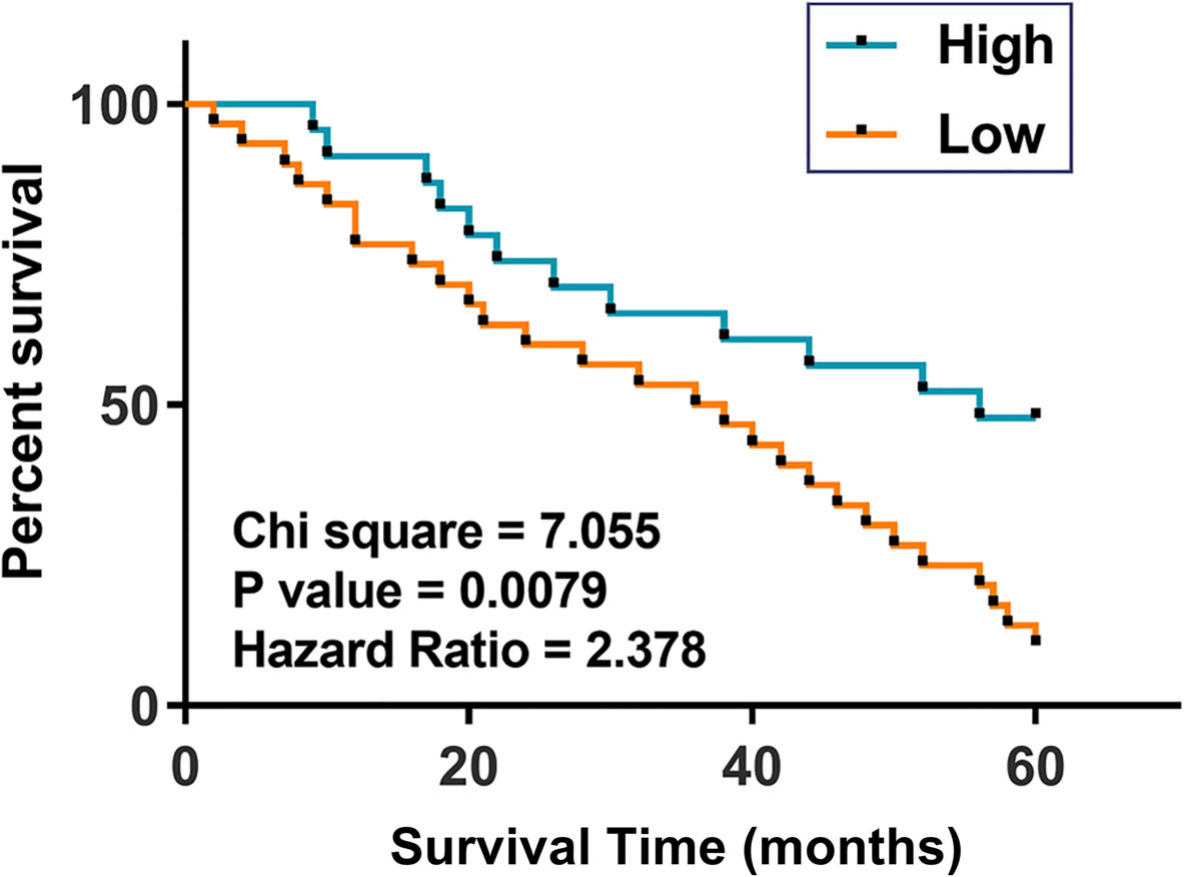
The expression levels of WT1-AS predicted NSCLC survival. Survival curve analysis was performed based on the 5-year follow-up study. Survival curves for low (< 3.9, *n* = 34) and high (≥ 3.9, *n* = 32) WT1-AS level groups were plotted and compared by log-rank test

Using the expression data of WT1-AS in NSCLC tissues before therapies, the above-average group was defined as high and sub-average group was defined as low. NSCLC patients were grouped into low (< 3.9, *n* = 34) and high (≥ 3.9, *n* = 32) WT1-AS level groups. Survival curves were plotted and compared using the methods aforementioned. Compared to patients in high in WT1-AS level group, low WT1-AS level group exhibited significantly lower overall survival rate (Fig. 2).

### The expression of UCA1 was inversely correlated with WT1-AS in NSCLC tissues

**Fig. 3:**
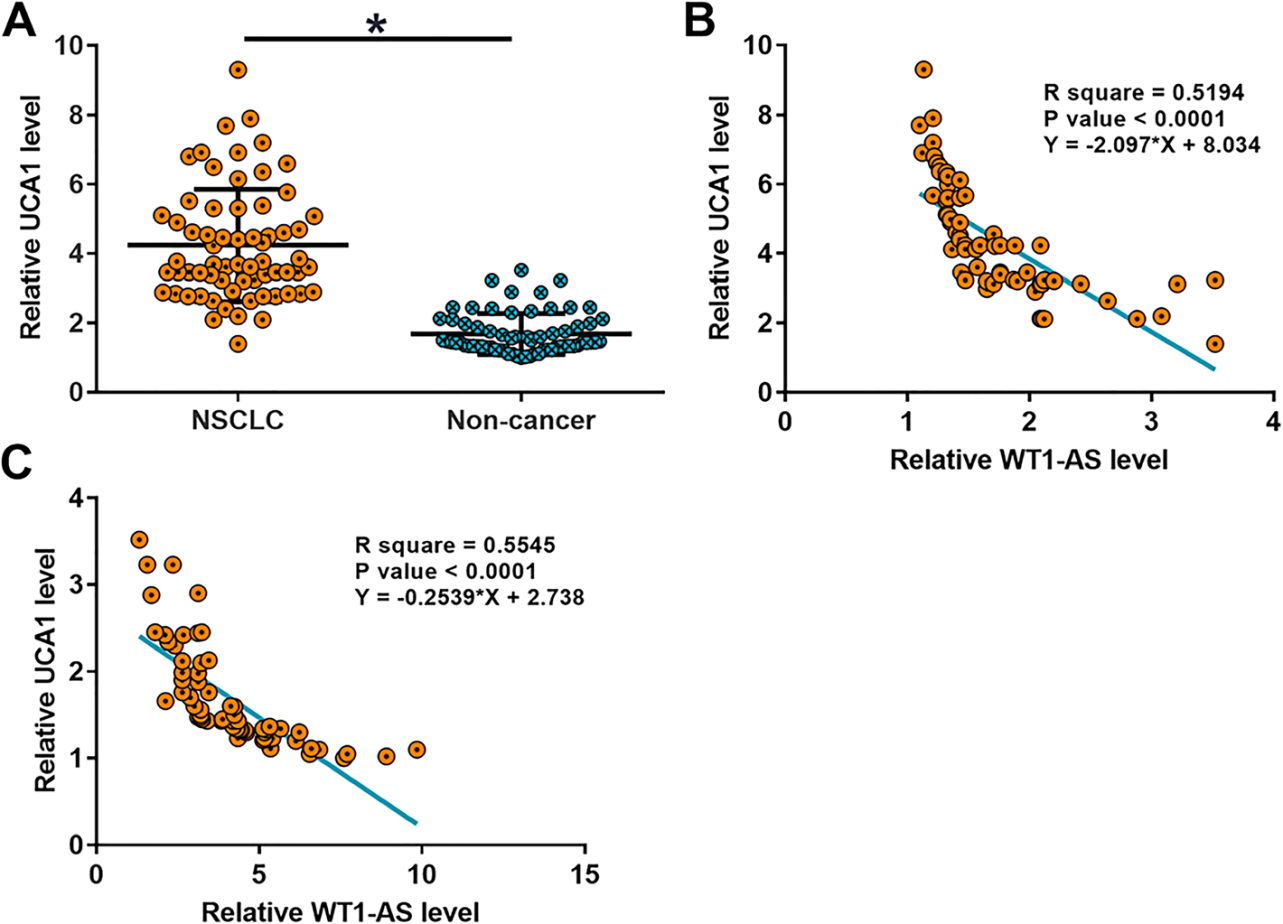
UCA1 was inversely correlated with WT1-AS in NSCLC tissues. UCA1 expression levels measured by RT-qPCR (**a**), (*, *p* < 0.05). Linear regression was performed to analyze the correlations between UCA1 and WT1-AS across NSCNC tissues (**b**) and non-cancer tissues (**c**)

The expression of UCA1 in paired tissues was also determined. Compared to non-tumor tissues, NSCLC tissues exhibited significant higher expression levels of UCA1 in non-cancer tissues (Fig. 3a, *p* < 0.05). Correlation analysis showed that the expression of UCA1 and WT1-AS were inversely and significantly correlated in both NSCNC tissues (Fig. 3b) and non-cancer tissues (Fig. 3c).

### WT1-AS suppressed NSCLC cell proliferation by downregulating UCA1

**Fig. 4:**
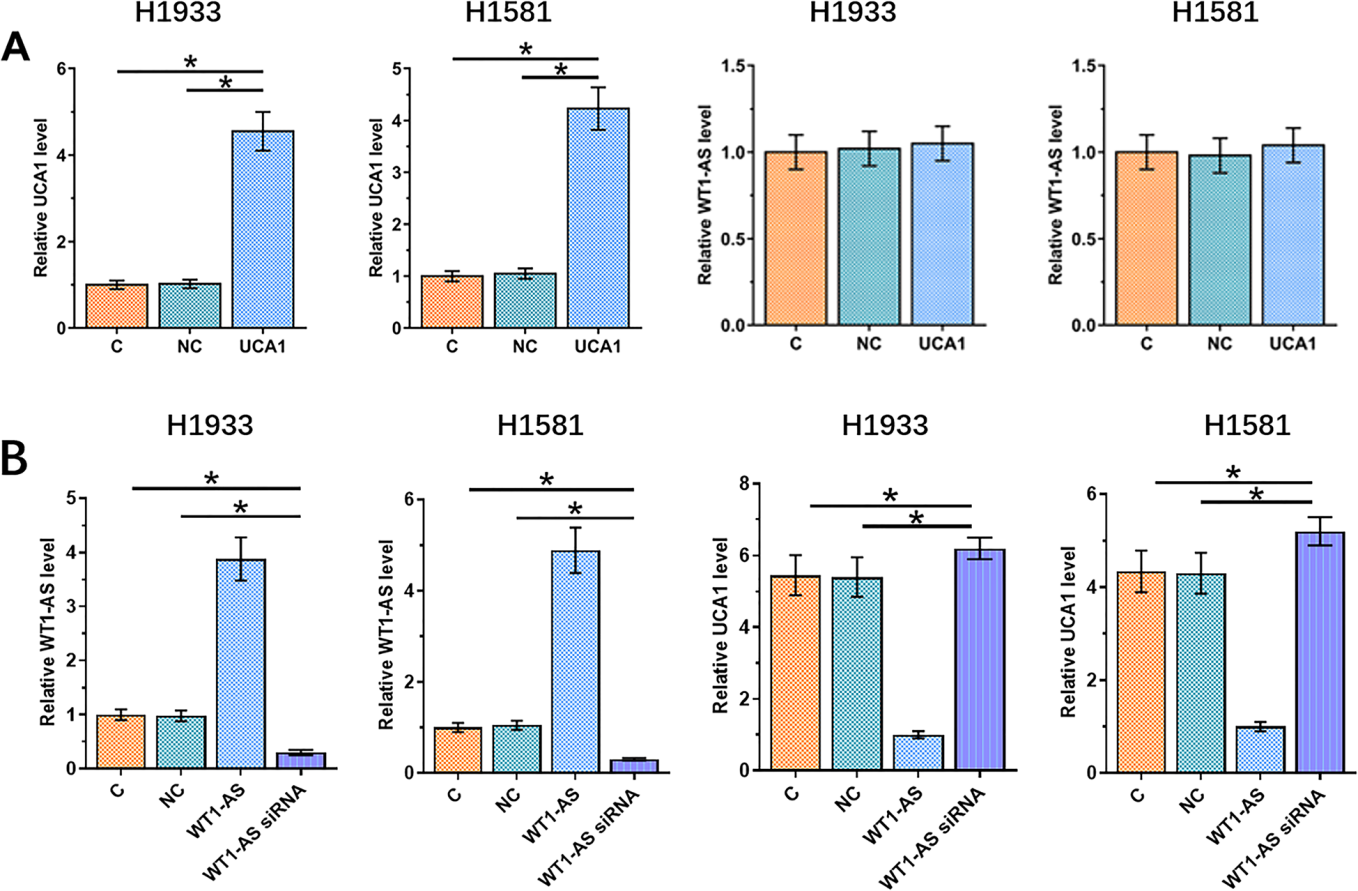
WT1-AS downregulated UCA1 to inhibit NSCLC cell invasion and migration. H1993 and H1581 cells were transfected with WT1-AS and UCA1 expression vectors and expression levels of UCA1 (left panel) and WT1-AS (right panel) were significantly increased at 24 h post-transfection comparing to NC and C groups (**a**). Compared to two controls (left panel), cells with UCA1 overexpression showed no significantly altered expression of WT1-AS, while WT1-AS overexpression led to inhibited UCA1 expression. And when WT1-AS was silenced, the expression of UCA1 was increased (right panel, **b**). NC, cells transfected with empty vector; C, untransfected cells. *, *p* < 0.05

**Fig. 5:**
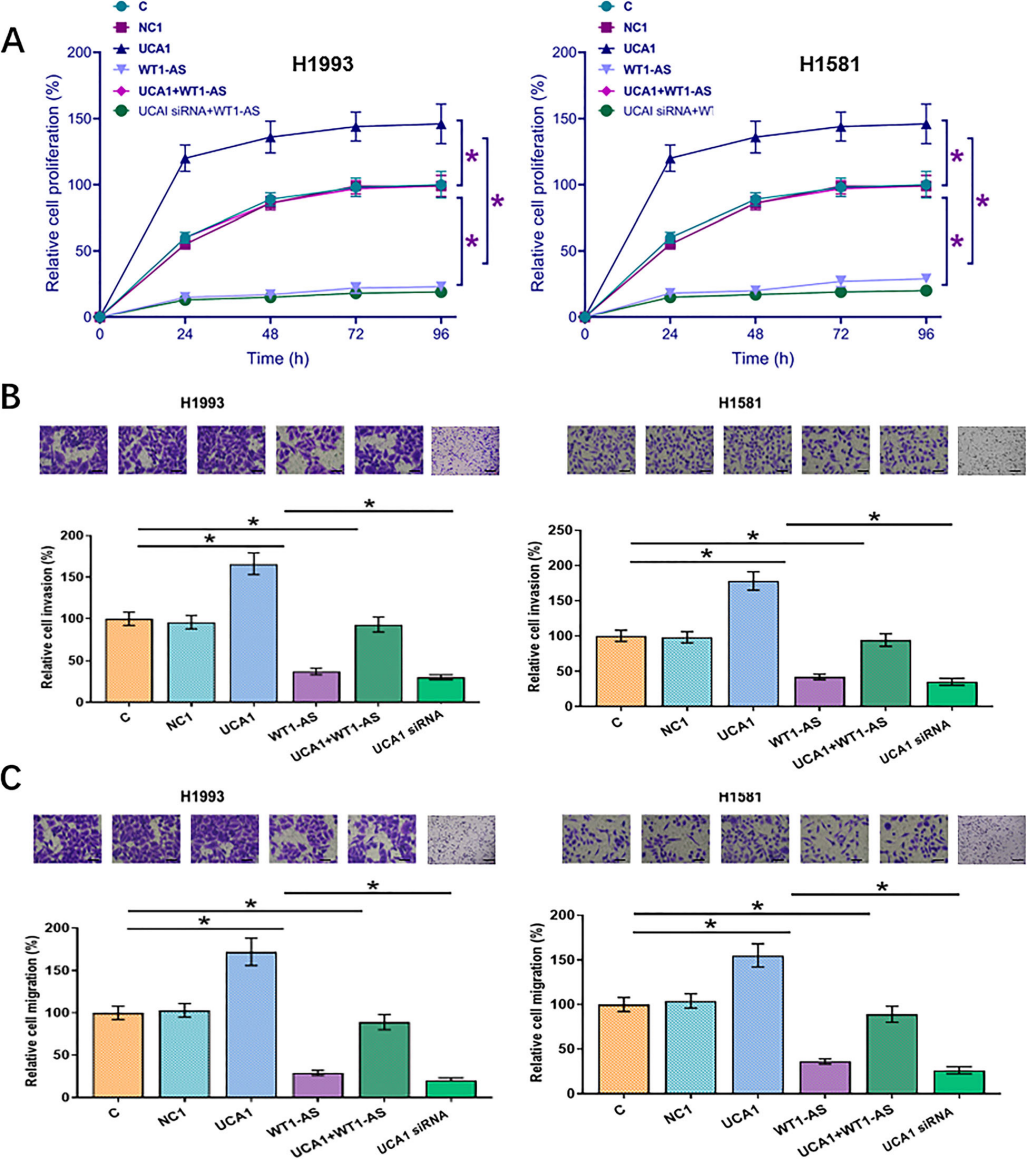
WT1-AS downregulated UCA1 to inhibit NSCLC cell proliferation, invasion and migration. WT1-AS downregulated UCA1 to suppress NSCLC cell proliferation (**a**). Cell proliferation assay was performed to assess the effects of the overexpression of WT1-AS and UCA1 on the proliferation of NSCLC cells. Mean values of 3 biological replicates are presented (*p < 0.05). Transwell assay showed that UCA1 overexpression promoted, while WT1-AS overexpression inhibited invasion (**b**) and migration (**c**) of NSCLC cells. In addition, the role of WT1-AS overexpression was inhibited by the overexpression of UCA1. Mean values of 3 biological replicates are presented. Scale bar represent 100 μm. NC, cells transfected with empty vector; C, untransfected cell. *, *p* < 0.05

WT1-AS or UCA1 expression vector was transfected into H1993 and H1581 cells. Transfections were confirmed at 24h post-transfection (Fig. 4a, *p *<0.05). UCA1 overexpression failed to affect WT1-AS, while WT1-AS overexpression led to inhibited UCA1 expression (Fig. 4b, *p *<0.05). Cell proliferation assay was performed to assess the effects of overexpression of WT1-AS and UCA1 on the proliferation of NSCLC cells. Compared to C and NC groups, cell proliferation assay showed that overexpression of WT1-AS led to decreased rates of cell proliferation, while overexpression of UCA1 led to increased proliferation of NSCLC cells. In addition, overexpression of UCA1 attenuated the effects of overexpression of WT1-AS. Moreover, silencing of UCAI decreased cell proliferation rate of the NSCLC cells (Fig. 5a *p* < 0.05).

### WT1-AS downregulated UCA1 to inhibit NSCLC cell invasion and migration by suppressing EMT and promoting the expression of p53

**Fig. 6:**
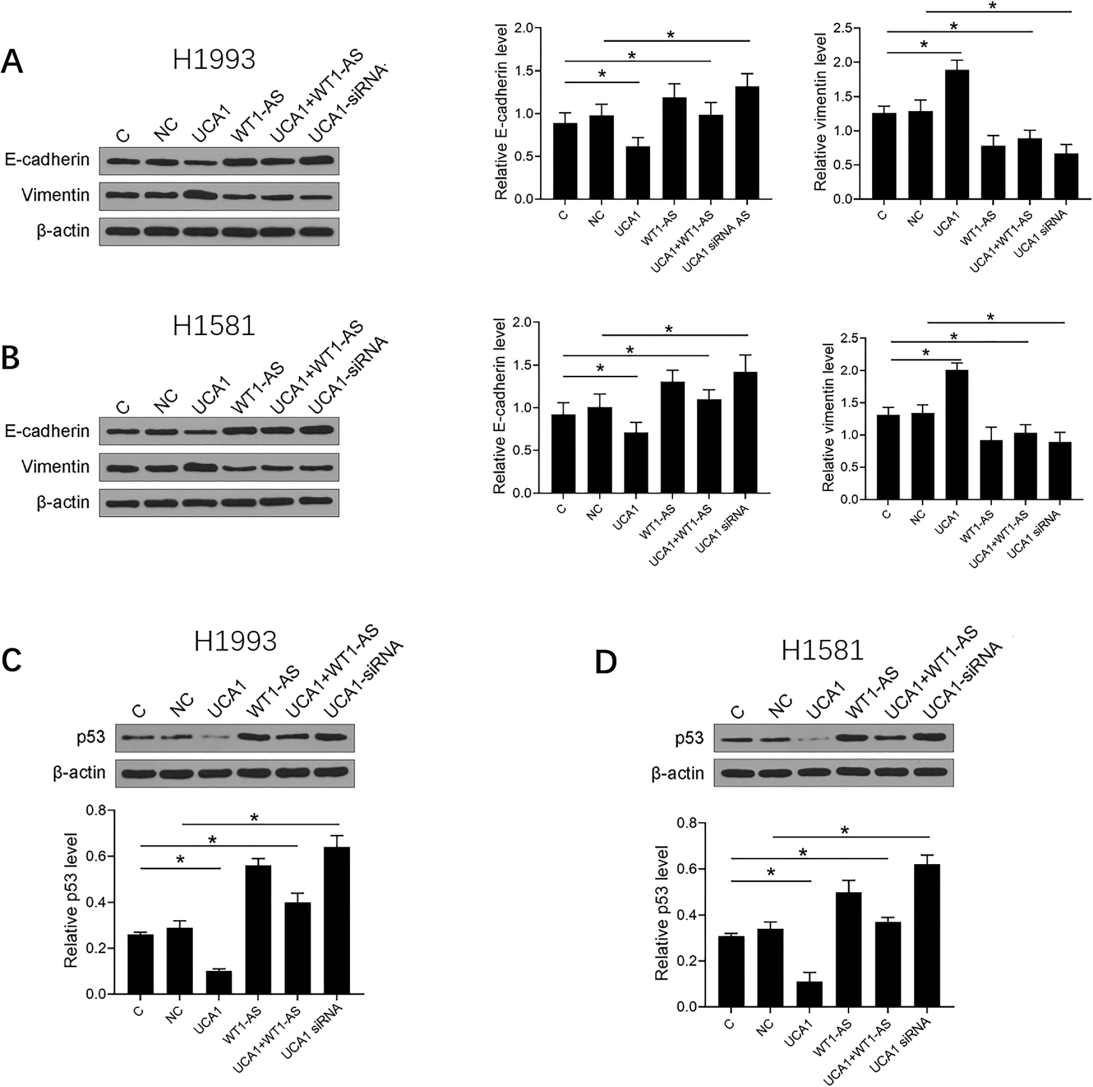
WT1-AS downregulated UCA1 to inhibit NSCLC cell invasion and migration through suppressing EMT and promoting the expression of p53. The effects of WT1-AS and miR-203 overexpression on the expression of E-cadherin, vimentin (**a** and **b**) and p53 (**c**) analyzed by Western blot, respectively. Mean values of 3 biological replicates are presented. NC, cells transfected with empty vector; C, untransfected cells. *p < 0.05

Transwell assay data showed that overexpression of UCA1 resulted in increased, while overexpression of WT1-AS resulted in decreased invasion (Fig. 5b) and migration (Fig. 5c) of NSCLC cells (*p* < 0.05). In addition, the role of overexpression of WT1-AS was inhibited by the overexpression of UCA1 (*p* < 0.05). Moreover, we also explored the function of WT1-AS and UCA1 in the level of EMT, the expression of E-cadherin and vimentin indicated that the decreased influence of UCA1 overexpression in EMT was counteracted by synchronous introduction of WT1-AS overexpression in H1993 cell (Fig. 6a) and H1581 cell (Fig. 6b). Meanwhile, we detected the expression of p53 to further demonstrate that WT1-AS downregulated UCA1 to inhibit NSCLC cell. And we found that overexpression of WT1-AS overexpression could promote the expression of p53 and when UCA1 was silenced, the expression levels of p53 were increased (Fig. 6c and d), the original, uncropped blots are shown in Supplementary figure-1. In summary, WT1-AS downregulated UCA1 to inhibit NSCLC cell invasion and migration by suppressing EMT and promoting the expression of p53.

## Discussion

The role of WT1-AS in NSCLC was investigated in this study. We found that downregulation of WT1-AS in NSCLC was correlated with the poor survival of NSCLC patients. In addition, overexpression of WT1-AS may inhibit NSCLC cell invasion and migration through the downregulation of UCA1.

The progression of NSCLC requires the involvement of lncRNAs [13]. For instance, lncRNA PVT1 is upregulated in NSCLC and promotes tumorigenesis [14]. In contrast, lncRNA MEG3 is downregulated in NSCLC and interacts with p53 to induce cancer cell apoptosis and inhibit cancer cell proliferation [15]. UCA is a well-characterized oncogenic lncRNA in different types of cancer including NSCLC [12]. Consistently, we also showed that upregulation of UCA1 in NSCLC tissues and its enhancing effects on cancer cell invasion and migration [12]. Based on our knowledge, the expression pattern of WT1-AS has only been characterized in gastric cancer [13]. In this study, we found that WT1-AS was downregulated in NSCLC and it inhibited the invasion and migration of cancer cells. Our data suggest that WT1-AS is a tumor suppressor in NSCLC.

Accurate prognosis assignment provided guidance for the determination of treatment strategies and post-treatment care [16]. This study showed that low expression levels of WT1-AS in NSCLC tissues before therapies predicted the poor survival of NSCLC patients. Therefore, measuring the expression levels of WT1-AS before treatment may assist the prognosis of NSCLC.

LncRNAs play a tumor-suppressive or oncogenic role in cancer biology by regulating downstream cancer-related pathways and other non-coding RNAs, such as miRNAs [17, 18]. However, the interactions between different lncRNAs have not been well studied. Our study showed that WT1-AS downregulated the expression of UCA1 in NSCLC cells, and decreased cell proliferation and ETM, thereby inhibiting NSCLC cell invasion and migration. Our study provided new insights into the pathogenesis of NSCLC. However, more studies are needed to further investigate the mechanism mediating the interactions between UCA1 and WT1-AS.

It is worth noting that a larger sample size is needed to further improve the authenticity of the conclusions. In this study, our study only included in vitro cell experiments. However, whether these tumors have deletions at the 11p13 chromosomal locus, the potential silencing by epigenetic mechanisms, or possibly some other reasons causing the lower levels of WT1-AS in NSCLC relative to normal lung is still unknown. Therefore, in vivo experiments are needed to test the roles of the interaction between UCA1 and WT1-AS in tumor progression as well as the potential reasons underlying the lower levels of WT1-AS in NSCLC relative to normal lung.

## Conclusions

In conclusion, WT1-AS is downregulated in NSCLC and WT1-AS overexpression inhibit NSCLC cell invasion and migration by downregulating UCA1.

## Supplementary Information

**Additional file 1: Figure S1.** The original, uncropped blots for Fig. 6.

## Data Availability

The datasets used and/or analyzed during the current study are available from the corresponding author on reasonable request.

## Abbreviations

NSCLC: Non-small cell lung cancer
lncRNAs: long non-coding RNAs
