# Supplementary figures and images for: LncRNA WT1-AS downregulates lncRNA UCA1 to suppress non-small cell lung cancer and predicts poor survival
Source: BMC Cancer. 2021 Jan 29;21:104. doi: 10.1186/s12885-020-07767-4 (PMC7844960; doi:10.1186/s12885-020-07767-4)

Figure 6A

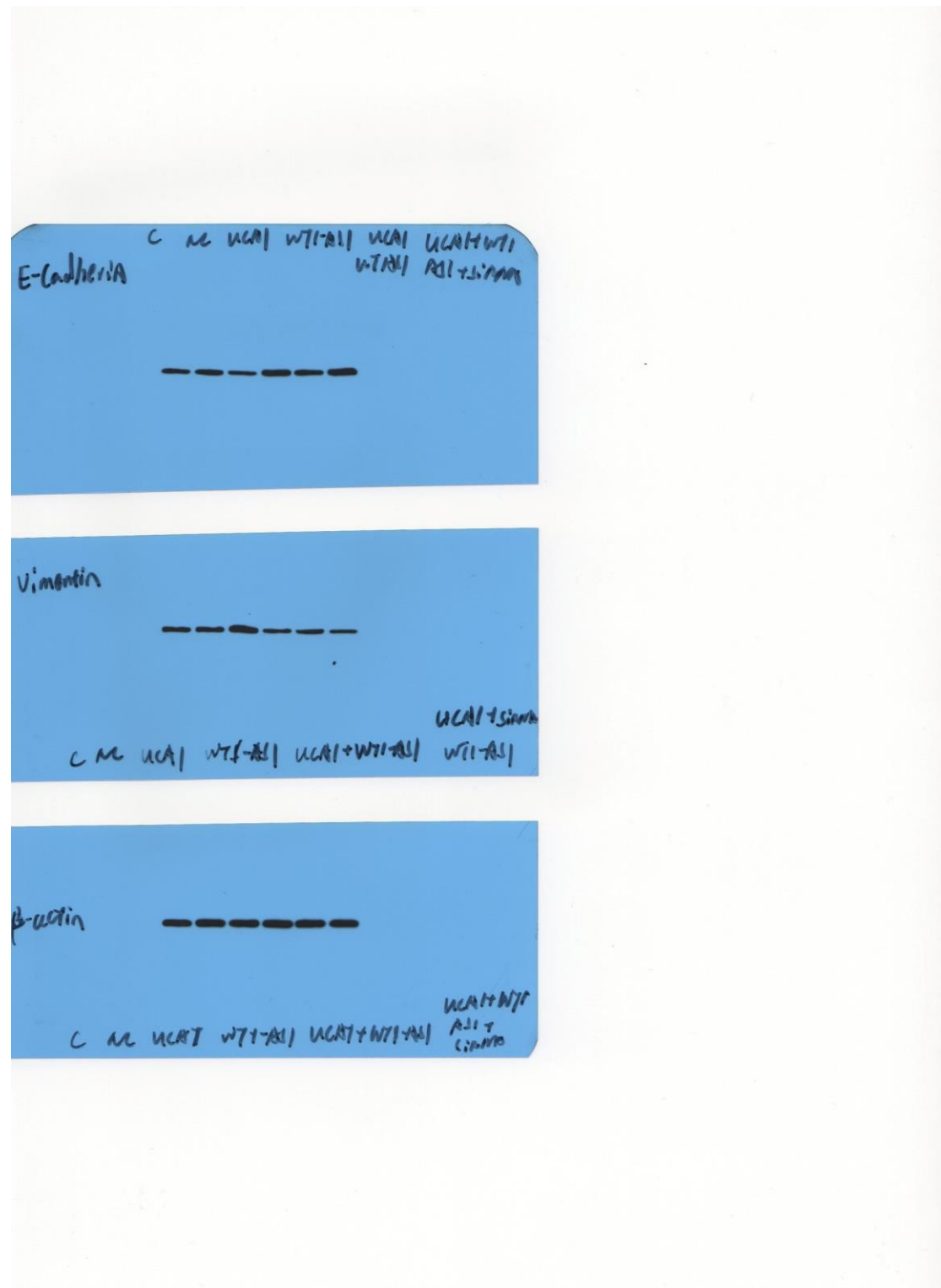

Figure 6B

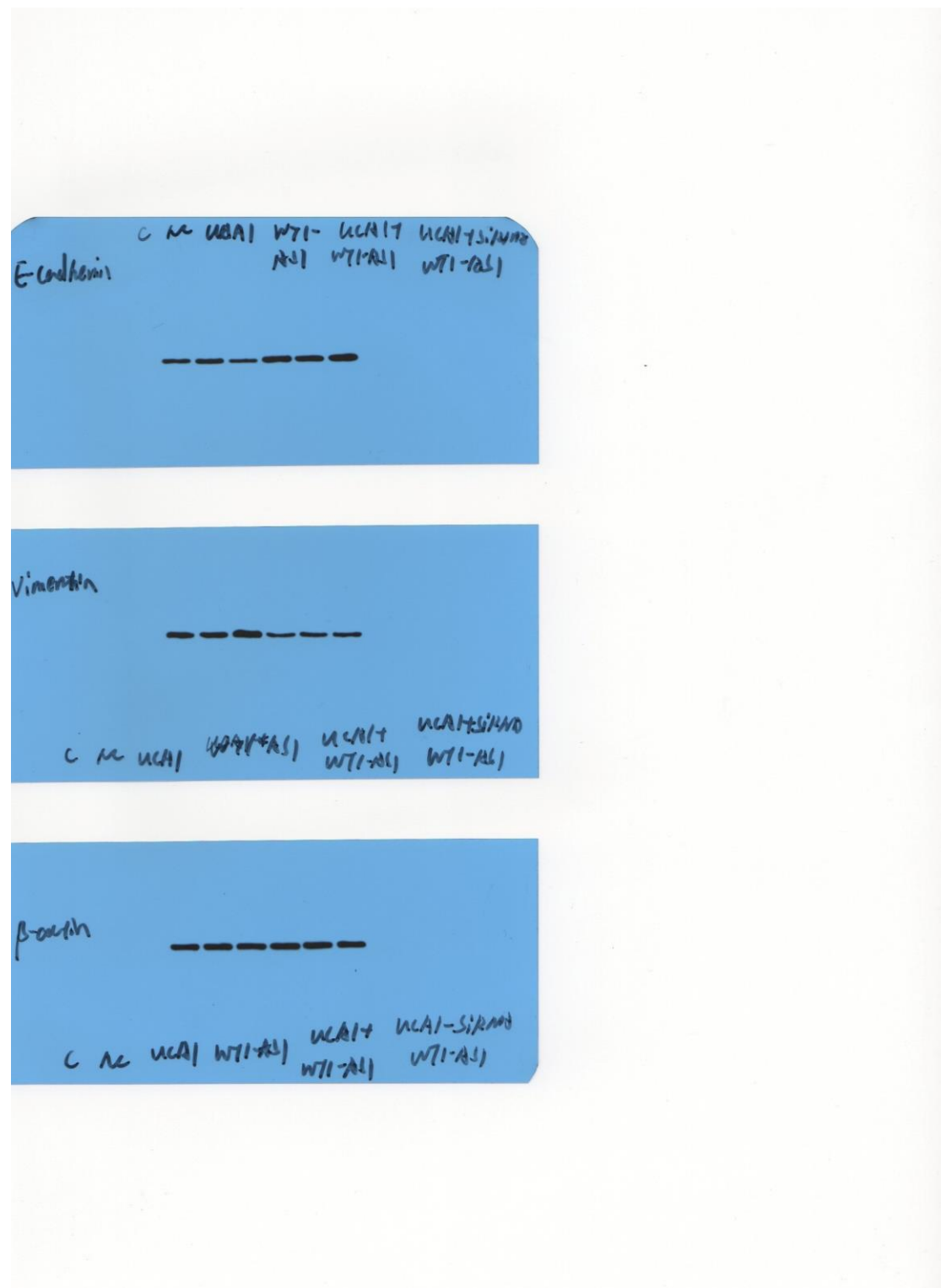

Figure 6C

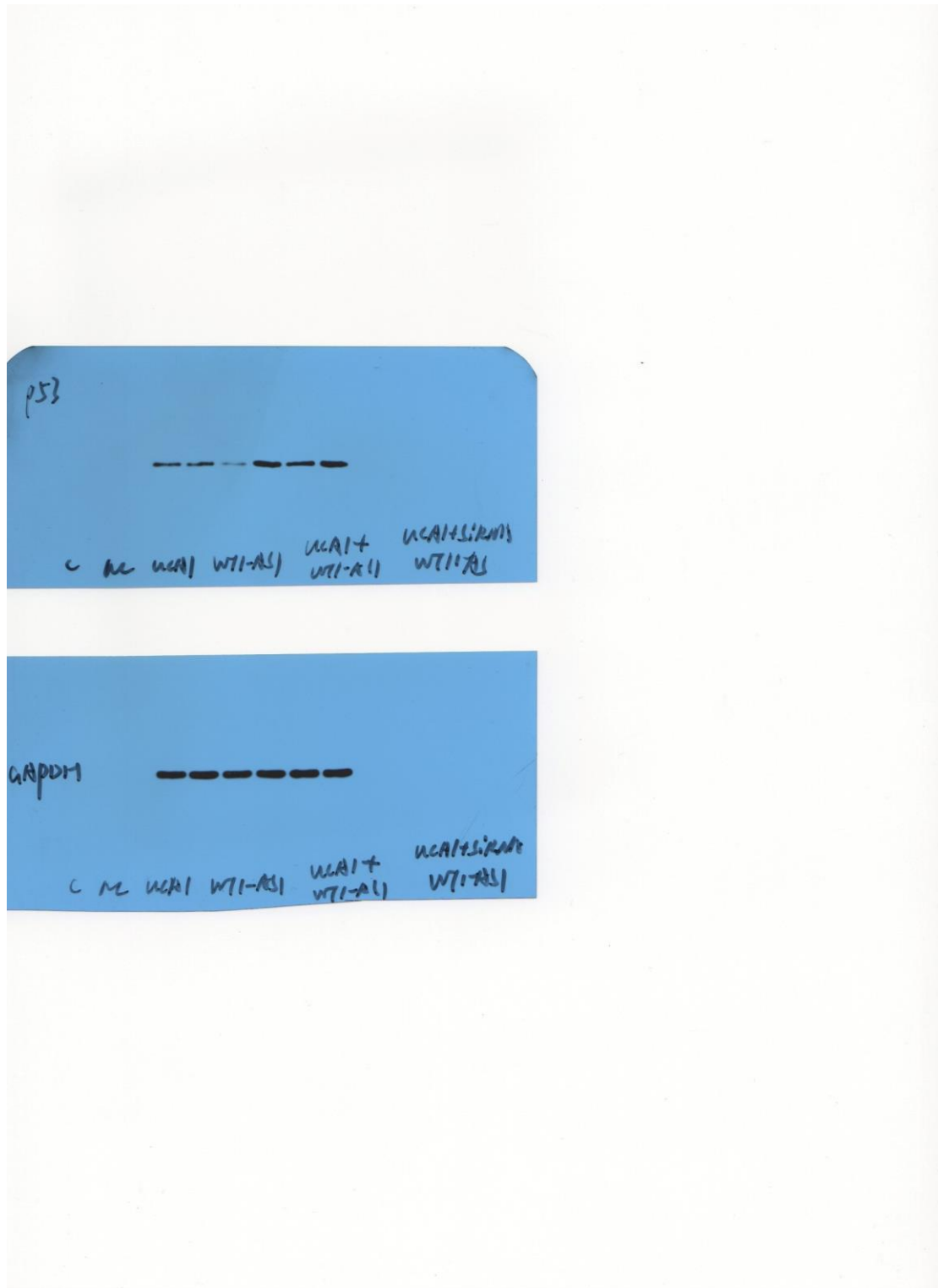

Figure 6D

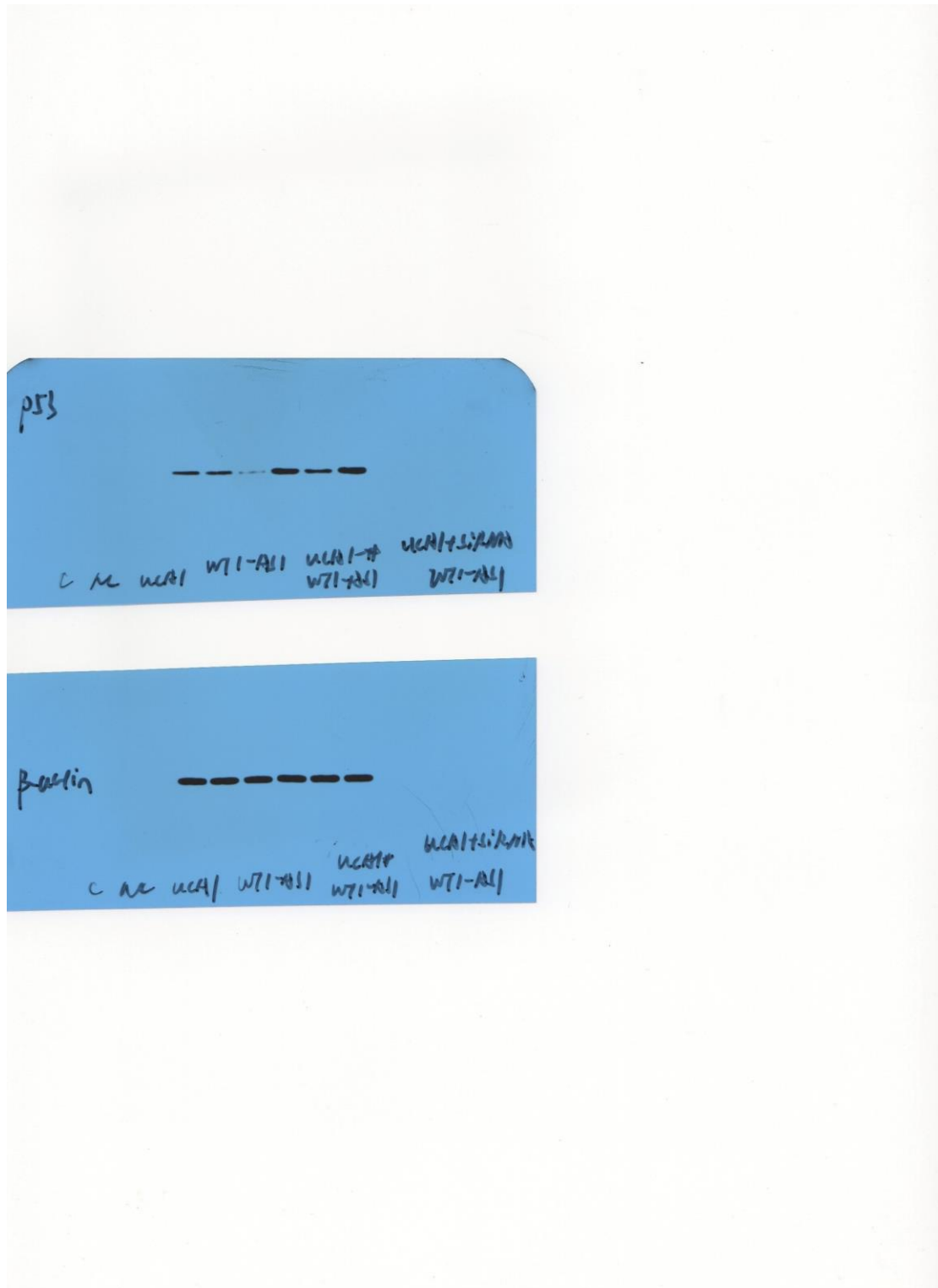

Supplement: Supplementary file 1 — Additional file 1: Figure S1. The original, uncropped blots for Fig. 6. [file 12885_2020_7767_MOESM1_ESM.pdf]
